# Serum concentration impacts myosin heavy chain expression but not cellular respiration in human LHCN‐M2 myoblasts undergoing differentiation

**DOI:** 10.1113/EP090564

**Published:** 2023-01-09

**Authors:** Mark C. Turner, Ryan Brett, Amarjit Saini, Claire E. Stewart, Derek Renshaw

**Affiliations:** ^1^ Centre for Sport, Exercise and Life Sciences Institute for Health and Wellbeing Coventry University Coventry UK; ^2^ Division of Clinical Physiology Department of Laboratory Medicine Karolinska, Institutet Karolinska University Hospital Huddinge Stockholm Sweden; ^3^ Research Institute of Sport and Exercise Science Life Sciences Building Liverpool John Moores University Liverpool UK

**Keywords:** cell culture, cell respiration, *ex vivo*, human serum, myogenesis

## Abstract

The human LHCN‐M2 myoblast cell line has the potential to be used to investigate skeletal muscle development and metabolism. Experiments were performed to determine how different concentrations of human serum affect myogenic differentiation and mitochondrial function of LHCN‐M2 cells. LHCN‐M2 myoblasts were differentiated in serum‐free medium, 0.5% or 2% human serum for 5 and 10 days. Myotube formation was assessed by immunofluorescence staining of myosin heavy chain (MHC) and molecularly by mRNA expression of *Myogenic differentiation 1* (*MYOD1*) and *Myoregulatory factor 5* (*MYF5*). Following differentiation, mitochondrial function was assessed to establish the impact of serum concentration on mitochondrial function. Time in differentiation increased mRNA expression of *MYOD1* (day 5, 6.58 ± 1.33‐fold; and day 10, 4.28 ± 1.71‐fold) (*P* = 0.012), while suppressing the expression of *MYF5* (day 5, 0.21 ± 0.11‐fold; and day 10, 0.06 ± 0.03‐fold) (*P* = 0.001), regardless of the serum concentration. Higher serum concentrations increased MHC area (serum free, 11.92 ± 0.85%; 0.5%, 23.10 ± 5.82%; 2%, 43.94 ± 8.92%) (*P* = 0.001). Absolute basal respiration approached significance (*P* = 0.06) with significant differences in baseline oxygen consumption rate (*P* = 0.025) and proton leak (*P* = 0.006) when differentiated in 2% human serum, but these were not different between conditions when normalised to total protein. Our findings show that increasing concentrations of serum of LHCN‐M2 skeletal muscle cells into multinucleated myotubes, but this does not affect relative mitochondrial function.

## Abstract (highlights)

- **What is the central question of this study?**
  Does the concentration of human serum affect skeletal muscle differentiation and cellular respiration of LHCN‐M2 myoblasts?
- **What is the main finding and its importance?**
  The concentration of serum used to differentiate LHCN‐M2 skeletal muscle cells impacts the coverage of myosin heavy chain, a marker of terminally differentiated myotubes. Normalisation of mitochondrial function data to total protein negates the differences observed in absolute values, which differ as a result of increased protein content when differentiation occurs with increasing concentration of serum.

## 1 INTRODUCTION

Skeletal muscle accounts for ∼40% of lean body mass in young, healthy adults and is important in the maintenance of overall health through the human lifespan (McLeod et al., 2016). Consequently, congenital defects in structural proteins, a reduction in skeletal muscle mass and dysfunction in metabolic capabilities (e.g., with age, disuse, diseases, e.g., muscular dystrophy, cancer cachexia and diabetes *mellitus*) all impact negatively on function and quality of life (Bouzakri et al., 2005; Fearon et al., 2012; Morgan & Partridge, 2020). Therefore, understanding fundamental skeletal muscle physiology and pathophysiology is paramount in identifying disease onset and progression as well as establishing effective interventions for patients with congenital or non‐communicable diseases where skeletal muscle health is reduced (Savikj & Zierath, 2020).

To understand and influence the regulators of skeletal muscle physiology and pathophysiology, many in vitro studies are undertaken utilising rodent cell lines (L6 rat or C2C12 mouse myoblasts), donor‐derived primary skeletal muscle cells from biopsies and pluripotent stem cells (Aas et al., 2013; Abdelmoez et al., 2020; Kodaka et al., 2017), all of which have their place within the field of skeletal muscle research. While primary human skeletal muscle cells have their advantages for understanding the innate characteristics of the donor and have greater relevance to human diseases, their finite replication potential can provide challenges when investigating the impact of exogenous factors such as nutritional status or electrical pulse stimulation on human skeletal muscle cell development and metabolism (Aas et al., 2013).

The LHCN‐M2 myoblast cell line, which is derived from the pectoralis major muscle of a male donor and was developed by transduction with retroviral vectors containing the *cdk‐4* and *hTERT* gene, is able to differentiate into multi‐nucleated myotubes and perform as well as non‐immortal skeletal muscle cells (Zhu et al., 2007). Consequently, LHCN‐M2 myoblasts provide an immortalised human skeletal muscle cell line to investigate skeletal muscle development, physiology and metabolism in vitro (Houghton et al., 2019; Salvadó et al., 2014, 2013; Vitucci et al., 2018).

Supplementation of the culture medium with serum, including human serum (Vitucci et al., 2018), or with serum‐free supplements, has been used to induce differentiation of skeletal muscle cells with varying responses (Lawson & Purslow, 2000; Saini et al., 2018). Using C2C12 and L6 myoblasts, several experiments have demonstrated the value of investigating potential influences of exogenous factors in human serum on skeletal muscle development, protein synthesis and mitochondrial function (Allen et al., 2021, 2022; Bruckbauer & Zemel, 2011; Carson et al., 2018; Cogan et al., 2019). However, the impact of serum concentration on differentiation and mitochondrial function in human LHCN‐M2 skeletal muscle cells is limited (Houghton et al., 2019) despite the close relationship between the two in human and rodent skeletal muscle cells (Hoffmann et al., 2018; Remels et al., 2010).

Using a human skeletal muscle cell line such as LHCN‐M2 in conjunction with a human serum microenvironment has the potential to provide a human‐centric in vitro model which will enhance the translational potential to investigate the cellular mechanisms in a multitude of physiological and pathophysiological conditions which impact upon skeletal muscle. Therefore, the purpose of these experiments was to determine how different concentrations of human serum affect LNCN‐M2 skeletal muscle differentiation and the subsequent impact on mitochondrial function. It was hypothesised that differentiation with increasing concentrations of serum would enhance myogenesis and mitochondrial function in LHCN‐M2 skeletal muscle myoblast cells.

## 2 METHODS

### 2.1 Cell culture

LHCN‐M2 human skeletal muscle myoblasts (passage 3–10) (Evercyte, Vienna, Austria) are an established myogenic cell line which was obtained from pectoralis major muscle tissue of a male donor in accordance with ethical procedures and the *Declaration of Helsinki* as outlined when the myoblasts were initially established (Zhu et al., 2007). LHCN‐M2 myoblasts were grown in four parts Dulbecco's modified Eagle's medium (DMEM; 4.5 mg/ml glucose) (Thermo Fisher Scientific, Paisley, UK, cat. no. 11960044) to one part medium 199 (M199; VWR, Lutterworth, UK, L0356), 15% fetal bovine serum (FBS) (BioSera, Nuaille, France, cat. no. FB‐1001/500), 2 mM GlutaMAX (Thermo Fisher Scientific, cat. no. 35050‐038), 20 mM HEPES (Thermo Fisher Scientific, cat. no. 10204932), 0.03 μg/ml zinc sulfate (Sigma‐Aldrich, Gillingham, UK, cat. no. Z0251), 1.4 μg/ml vitamin B12 (Sigma‐Aldrich, cat. no. V2876), 0.055 μg/ml dexamethasone (Sigma‐Aldrich, cat. no. D4902), 2.5 ng/ml hepatocyte growth factor (HGF) (Peprotech, London, UK cat. no. 100‐39H), 10 ng/ml fibroblast growth factor–basic (bFGF) (Peprotech, cat. no. 100‐18B). Cells were seeded at 1200 cells/cm^2^ on 0.2% porcine type B gelatin (Sigma‐Aldrich, G1393)‐coated plates until 90% confluence was attained, at which point differentiation was induced for 10 days in a fusion medium containing 4:1 DMEM/M199 as outlined above, 20 mM HEPES, 0.03 μg/ml zinc sulphate, 1.4 μg/ml vitamin B12, 50 μg/ml apo‐transferrin (Sigma‐Aldrich, T1147), with 0% (serum free), 0.5% or 2% human serum in order to induce the formation of multi‐nucleated myotubes. The human serum was purchased commercially (cat no. H6914, lot no. SLBW5267; Sigma‐Aldrich) and was obtained from male donors who provided samples in Food and Drug Administration (FDA)‐licensed centres located in the USA. The serum composition, which is obtained from the certificate of analysis, is outlined in Table 1.

**TABLE 1:** Composition of commercially available human serum (Sigma‐Aldrich cat no.: H6914, lot no. SLBW5267) derived from a male (AB clotted whole blood) used in differentiation medium

Measure	Value
**Glucose**	95 mg/dl
**Cholesterol**	150 mg/dl
**Triglycerides**	102 mg/dl
**Sodium**	140 mEq/l
**Protein**	6.5%
**Iron**	89% (UG%)
**Osmolality**	294 mOsmol/kg H_2_O
**pH**	7.4

### 2.2 Immunofluorescence

Cells were washed in ice‐cold phosphate‐buffered saline (PBS) before being fixed in 3.7% paraformaldehyde (Sigma‐Aldrich). Samples were permeabilised and blocked with blocking solution (PBS, 5% goat serum, 0.2% Triton X‐100) for 1 h at room temperature before being incubated overnight at 4°C with mouse monoclonal anti‐myosin heavy chain, sarcomere (MHC) antibody at a concentration of 1:100 (MF 20 was deposited to the DSHB by D. A. Fischman; DSHB Hybridoma Product MF 20) diluted in PBS with 2% goat serum and 0.2% Triton X‐100. Samples were washed with PBS and incubated for 2 h at room temperature with secondary fluorescent conjugated anti‐mouse antibody (1:500, Thermo Fisher Scientific, Alexa Fluor 555 goat anti‐mouse) and nuclei were counterstained with Hoechst 33342 (1:1000 of 1 μg/ml stock) diluted in PBS with 2% goat serum and 0.2% Triton X‐100. Wells were washed with PBS and covered with PBS containing 10% glycerol prior to imaging.

Images were obtained using a Cytation 5 cell imaging multimode reader with Gen5+ software (Agilent Technologies, Stockport, UK) at ×10 magnification and exported as TIFF files for processing and analyses. Image analysis was conducted using the publicly available image processing software FIJI (https://fiji.sc/). Using a macro function, all images were converted to 8‐bit, smoothed, sharpened and despeckled prior to background subtraction (rolling ball radius of 100 pixels). Images were then thresholded (Huang method) to create a binary image, and MHC positive area was determined as a percentage of the total field of view. Thirteen to fifteen images per condition were analysed from three independent experiments.

### 2.3 RNA extraction, cDNA synthesis and qPCR analysis

RNA was extracted using TRI Reagent (Sigma‐Aldrich) according to the manufacturer's instructions followed by RNA clean up using Monarch RNA Cleanup Kit (New England Biolabs UK, Hitchin, UK). RNA concentrations were quantified using UV spectroscopy (NanoDrop, Thermo Fisher Scientific). cDNA synthesis was conducted using a Quantitect reverse transcription kit according to the manufacturer's instructions (Qiagen, Manchester, UK). Gene expression was analysed using Quantifast SYBR green qPCR chemistry (Qiagen) using a QuantStudio 7 Flex Real‐Time PCR System (Thermo Fisher Scientific). Each reaction consisted of 2 ng of cDNA in a final 20 μl reaction volume with master mixes made according to the manufacturer's instructions (Qiagen) using Quantitect Primer Assay for *Myogenic differentiation 1* (*MYOD1*; QT00209713) and *Myoregulatory factor 5* (*MYF5*; QT00027825), along with *RNA polymerase II subunit B* (*POLR2B*; QT00081774), which was not affected by differentiation and was subsequently used as an endogenous house‐keeping control gene. Fluorescence was detected after every cycle (40 cycles), and data, which were run in triplicate for each experimental condition and time point, were analysed using the ΔΔ*C*_t_ method (Schmittgen & Livak, 2008).

### 2.4 Seahorse metabolic analysis

Live cell metabolic analysis was used to investigate changes in cellular respiration using the Agilent Seahorse XFe96 Analyser (Agilent Technologies, Stockport, UK). LNCN‐M2 cells were seeded at 10,000 cells per well and cultured as outlined above. On day 10 of differentiation, the culture medium was removed, and differentiated myotubes were washed with Seahorse assay medium (DMEM with 1 mM pyruvate, 2 mM glutamine and 10 mM glucose) before being incubated for 1 h at 37°C in a non‐CO_2_ incubator in fresh assay medium. A mitochondrial stress test was performed by injecting 1.5 μM oligomycin, 1 μM FCCP and 0.5 μM rotenone and antimycin A. All compounds were reconstituted in a seahorse assay medium prior to loading into the injection ports. The assay consisted of 3 × 3 measurement cycles interspersed with 3 min, or 5 min for carbonyl cyanide‐*p*‐trifluoromethoxyphenylhydrazone (FCCP), of mixing between each injection. Upon completion of the assay, RIPA buffer containing protease and phosphatase inhibitors was used to lyse the cells in order to determine protein concentration by BCA assay, as outlined below, in order to normalise changes in cell respiration between conditions. Data were exported and analysed using the Agilent Seahorse Analytics online platform (https://seahorseanalytics.agilent.com/).

### 2.5 Protein quantification

Protein concentrations were quantified using a BCA protein assay (Thermo Fisher Scientific). Ten microlitres of sample or standard (0–2000 μg/ml) was mixed with 200 μl of the working reagent, shaken and incubated at 37°C for 30 min in the dark before absorbance was measured at 562 nm using a colorimetric plate reader (Biotek/Agilent Technologies, Stockport, UK).

### 2.6 Statistical analysis

Data are presented as means ± standard deviation from three to four independent (*n*) experiments using GraphPad Prism 9 (GraphPad Software Inc., San Diego, CA, USA). Statistical analysis was conducted using SPSS Statistics software (IBM Corp., Armonk, NY, USA) using ANOVA with Bonferroni *post hoc* test to determine statistical differences between conditions (*P* < 0.05).

## 3 RESULTS

### 3.1 Differentiation of LHCN cells induces the formation of multinucleated skeletal muscle myotubes

**FIGURE 1:**
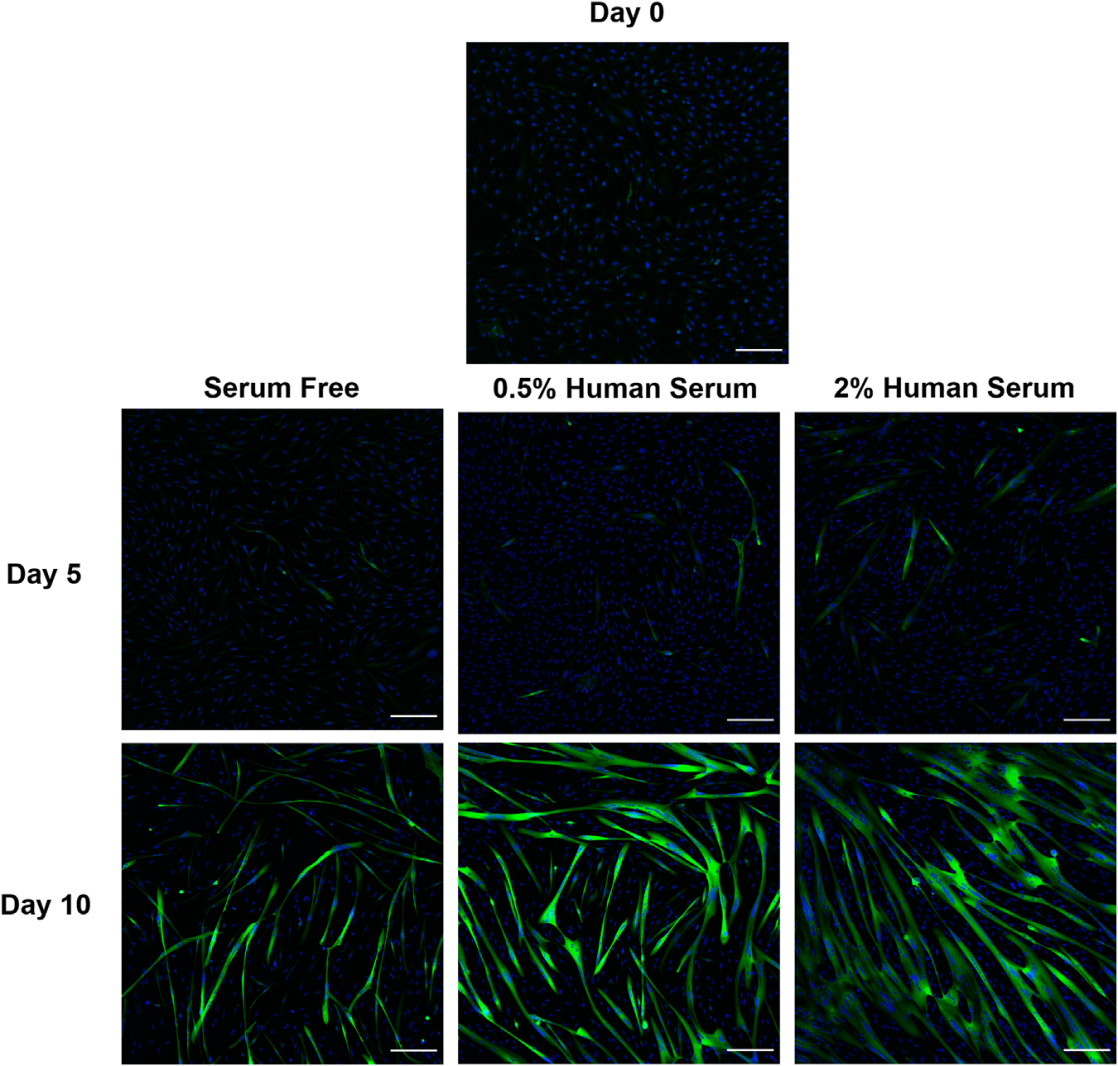
Representative immunofluorescence images of myosin heavy chain (MF 20) (green) and Hoechst 33342 (blue) in LHCN‐M2 myoblasts differentiated for 0, 5 and 10 days with serum‐free, 0.5% or 2% human serum medium. Scale bar: 200 μm

**FIGURE 2:**
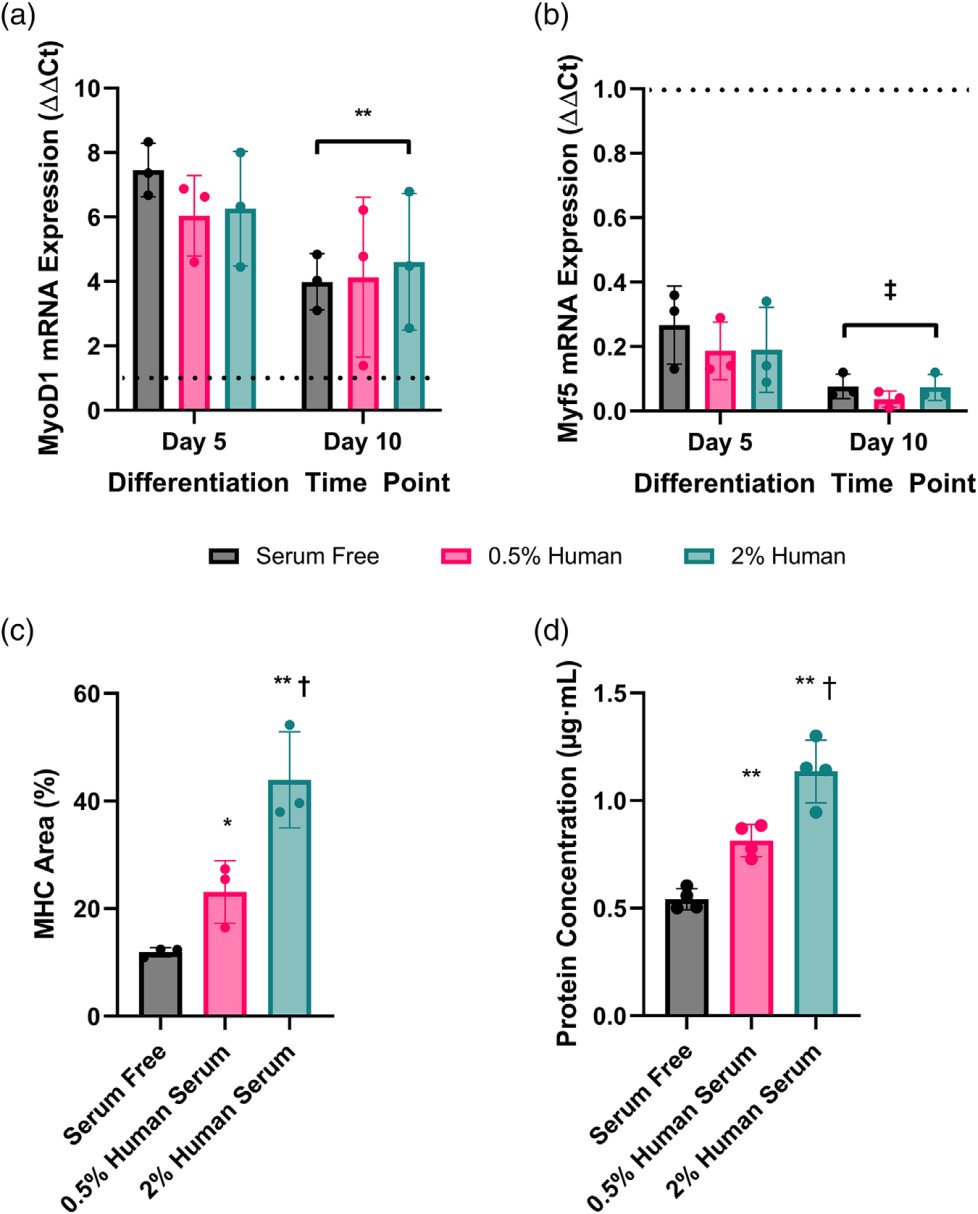
(a, b) LHCN‐M2 SKM cell mRNA expression of *MYOD1* (a) and *MYF5* (b) following 5 and 10 days’ differentiation in serum‐free, 0.5% or 2% human serum media. Significant effect for time versus D5: **P < 0.01, ‡*P* < 0.001. (c, d) Myosin heavy chain area (%) (c) and cell lysate protein concentrations (μg/μl) (d) following 10 days of differentiation with serum‐free, 0.5% or 2% human serum media. Significantly different versus serum‐free condition: *P < 0.05, **P < 0.01. Significantly different versus 0.5% human serum: †*P* < 0.05. Data are means ± standard deviation from 3–4 independent experiments

In order to assess the differentiation of LHCN‐M2 myoblasts to multi‐nucleated myotubes in response to low dose human serum, the mRNA expression of genes associated with skeletal muscle differentiation and the expression of pan‐MHC was measured from single myoblasts at the start of fusion into multinucleated myotubes (Figure 1). Relative to day 0, which had a cycle threshold (*C*_t_) of 28.19 ± 0.44 and 23.61 ± 0.53 for *MYOD1* and *MYF5*, respectively, the mRNA expression of genes which regulate myogenesis (*n* = 3) was either increased (*MYOD1*; day 5, 6.58 ± 0.45‐fold and day 10, 4.60 ± 0.51‐fold; *P* = 0.012) or reduced (*MYF5*; day 5, 0.21 ± 0.03‐fold and day 10, 0.06 ± 0.01‐fold; *P* = 0.001) over the course of differentiation (Figure 2a,b). However, neither *MYOD1* (*P* = 0.806) nor *MYF5* (*P* = 0.256) mRNA expression was affected by the concentration of serum. At day 10, MHC area (*n* = 3), as a percentage of the field of view, was greater following differentiation in 0.5% (23.11% ± 5.8%) and 2% (43.94% ± 8.92%) compared to serum‐free medium (11.92% ± 0.85%) (*P* = 0.001). In addition, MHC area was greater following differentiation in 2% compared to 0.5% human serum (Figure 2c; *P* = 0.021). This was mirrored with the increase in protein lysate concentrations (*n* = 4), which were greater in 0.5% (0.815 ± 0.075 μg/μl) (*P* = 0.01) and 2% human serum (1.140 ± 0.146; μg/μl) (*P* < 0.0001) compared to myotubes differentiated under serum‐free conditions (0.542 ± 0.049 μg/μl). Similar to MHC area, protein lysate concentrations were greater following differentiation in 2% compared to 0.5% human serum (*P* = 0.004, Figure 2d).

### 3.2 Mitochondrial function responses to differentiation in different concentrations of human serum

**FIGURE 3:**
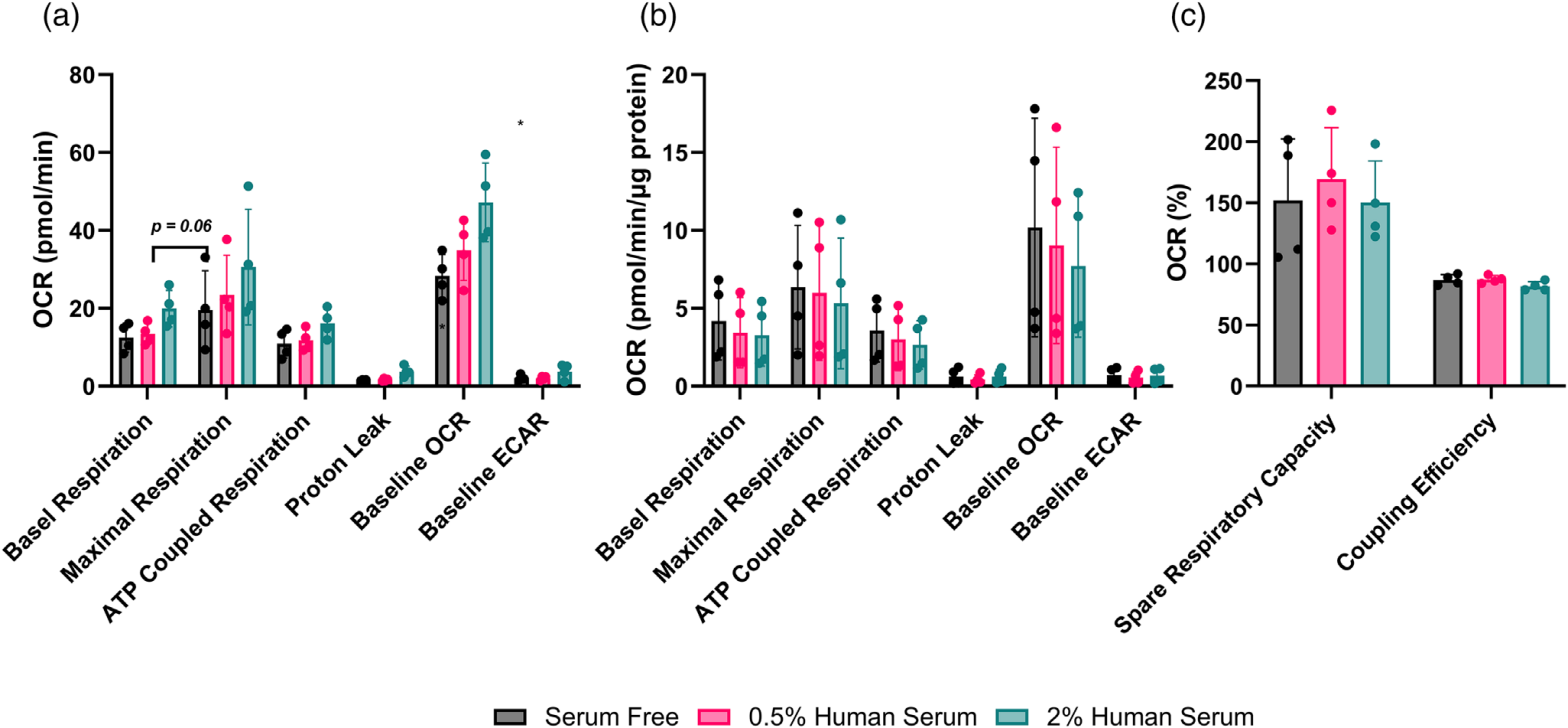
Mitochondrial function and content of LHCN‐M2 myoblasts differentiated in serum‐free, 0.5% or 2% human serum media. (a) Absolute mitochondrial function (pmol/min), (b) relative mitochondrial function (pmol/min/μg protein) and (c) spare respiratory capacity and coupling efficiency (% OCR). Data are means ± standard deviation from 3–4 independent experiments. Significantly different versus serum‐free condition: *P < 0.05. Significantly different versus 0.5% human serum: †*P* < 0.05

The impact of different concentrations of serum on mitochondrial function was assessed in LHCN‐M2 myotubes at day 10 of differentiation (*n* = 4). Analysis of absolute values showed myotubes differentiated in 2% human serum had a significantly higher basal oxygen consumption rate (OCR) compared to myotubes cultured in serum‐free medium (*P* = 0.025) with no differences in extracellular acidification rate (ECAR) (*P* = 0.127; Figure 3a). Analysis of mitochondrial function showed there to be higher basal respiration (*P* = 0.06) and significantly higher proton leak in myotubes cultured in 2% human serum compared to 0.5% human serum (*P* = 0.015) and serum‐free conditions (*P* = 0.011; Figure 3a); however, there were no differences in maximal (*P* = 0.445) or ATP‐coupled respiration (*P* = 0.117) between conditions (Figure 3a). When normalised to total protein content, there were no differences in OCR (*P* = 0.850), ECAR (*P* = 0.858), basal (*P* = 0.833), maximal (*P* = 0.939) or ATP‐coupled respiration (*P* = 0.789) or proton leak (*P* = 0.752); Figure 3b). Finally, there were no significant differences in spare respiratory capacity (*P* = 0.787) or coupling efficiency (*P* = 0.118) between conditions (Figure 3c).

## 4 DISCUSSION

The purpose of these experiments was to establish how the concentration of human serum influences myogenic differentiation and metabolic function in LHCN‐M2 myoblasts, a human immortalised skeletal muscle cell line (Zhu et al., 2007). Several groups have used LHCN‐M2 myoblasts to investigate the cellular and molecular responses of human skeletal muscle using varying compositions of differentiation medium in order to induce the formation of multinucleated myotubes (Houghton et al., 2019; Toral‐Ojeda et al., 2018; Zhu et al., 2007).

Similar to previous research, our findings demonstrated the differentiation potential of LHCN‐M2 cells in the presence of human serum (Vitucci et al., 2018). However, progressing from the findings of Vitucci et al., our experiments found that the concentration of human serum had a significant impact on the extent of myosin heavy chain positivity. Arguably, these findings are not surprising based on previous studies on chicken embryo myocytes, established rodent cell lines and human skeletal muscle cells, which have all shown the concentration of serum to be an important factor in skeletal muscle differentiation (Carson & Booth, 1998; Cheng et al., 2014; Lawson & Purslow, 2000).

Experiments reported here demonstrated that differentiation in serum‐free or low human serum (0.5 or 2%) media does not alter the transcriptional activation of *MYOD1* or *MYF5*, which are involved in the myogenic programme (Bentzinger et al., 2012; Zammit, 2017). Our findings complement the findings of Vitucci et al. who have shown that low concentrations of human serum (0.5%) induce differentiation of LHCN‐M2 myoblasts cells (Vitucci et al., 2018), activating other genes which are integral in myogenic differentiation (Buckingham & Rigby, 2014). It could be speculated that the induction of genes such as *MYF5* is dependent on the reduction in factors contained within the culture medium irrespective of the serum concentration (Jarocha et al., 2014).

Mitochondrial function is important in skeletal muscle both in adaptive (e.g., exercise training) and maladaptive circumstances (e.g., sarcopenia) (Hood et al., 2019). Absolute values showed a significant difference in non‐mitochondrial respiration, with basal respiration approaching a statistical difference between serum‐free and 2% human serum media. However, when normalised to total protein, these were not different along with maximal respiration, ATP‐ coupled respiration, coupling efficiency or spare capacity. The increase in absolute levels of basal respiration mirror the increase in both protein content and coverage of pan‐MHC suggesting the differences in absolute values were not due to a reduction in function of the mitochondria. Our findings support the importance of the normalisation of mitochondrial respiration data when interrogating skeletal muscle differentiation, although normalisation to protein concentration is commonly used and is closely correlated with other measures of mitochondrial content (Acin‐Perez et al., 2020). However, we acknowledge the importance of considering other normalisation methods such as citrate synthase activity or mitochondrial DNA in determining mitochondrial content depending on the experimental design being proposed (Divakaruni & Jastroch, 2022; Schmidt et al., 2021).

Our experiments showed that while the concentration of human serum did not alter the mRNA expression of *MYOD1* and *MYF5*, it did have a significant effect upon the expression of myosin heavy chain area of skeletal muscle myotubes. The importance of serum components in skeletal muscle differentiation has been demonstrated in primary human skeletal muscle cells (Saini et al., 2018). Previous research has shown that while differentiation into multinucleated myotubes occurs in the absence of serum, the growth factors contained in serum are an important contributor to the differentiation and maturation of human skeletal muscle cells in culture (Saini et al., 2018). In addition, previous research using LHCN‐M2 cells has demonstrated enhanced differentiation through increased expression of mature muscle markers such as MHC with supplementation of trophic factors such as insulin‐like growth factor 1 and insulin which facilitate skeletal muscle differentiation (Guo et al., 2014; Saini et al., 2018; Toral‐Ojeda et al., 2018). Although the serum used in these experiments is not completely defined, the lower percentage area of MHC could be explained by the absence of trophic factors and proteins in serum‐free and 0.5% serum differentiation media.

In summary, our experiments show that despite our hypothesis that the concentration of human serum in the culture medium would affect the mitochondrial function of differentiated LHCN‐M2 skeletal muscle myotubes, the greatest effects were on the coverage of MHC as a marker of mature skeletal muscle, which is potentially attributed to the concentration of trophic factors within the serum. This has potential implications for the absolute basal and non‐mitochondrial oxygen consumption levels observed during the analysis of mitochondrial function but not when normalised to total protein content. Further work is required to establish the link with skeletal muscle differentiation in LHCN‐M2 cells.

## AUTHOR CONTRIBUTIONS

Mark C. Turner, Claire E. Stewart and Amarjit Saini conceived the experiments, Mark C. Turner and Ryan Brett conducted the experiments and analysis of the samples. Mark C. Turner, Ryan Brett, Amarjit Saini, Claire E. Stewart and Derek Renshaw interpreted the data and drafted the manuscript. All authors have read and approved the final version of this manuscript and agree to be accountable for all aspects of the work in ensuring that questions related to the accuracy or integrity of any part of the work are appropriately investigated and resolved. All persons designated as authors qualify for authorship, and all those who qualify for authorship are listed.

## CONFLICT OF INTEREST

The authors declare that they have no conflicts of interest.

## FUNDING

No funding was received for conducting this research.

## Supporting information

Statistical Summary Document

## Data Availability

The data that support the findings of this study are available from the corresponding author upon reasonable request.
